# Effects of the electrical excitation signal parameters on the geometry of an argon-based non-thermal atmospheric pressure plasma jet

**DOI:** 10.1186/1556-276X-9-697

**Published:** 2014-12-26

**Authors:** Mohamed Tahar Benabbas, Salah Sahli, Abdallah Benhamouda, Saida Rebiai

**Affiliations:** Microsystems and Instrumentation Laboratory, Department of Electronics, Faculty of Sciences of Technology, University of Constantine 1, 25017 Constantine, Algeria

**Keywords:** Atmospheric pressure plasma jet, Electrical discharge parameters, Plasma jet geometry, Applied voltage, Signal frequency

## Abstract

**Abstract:**

A non-thermal atmospheric pressure argon plasma jet for medical applications has been generated using a high-voltage pulse generator and a homemade dielectric barrier discharge (DBD) reactor with a cylindrical configuration. A plasma jet of about 6 cm of length has been created in argon gas at atmospheric pressure with an applied peak to peak voltage and a frequency of 10 kV and 50 kHz, respectively. The length and the shape of the created plasma jet were found to be strongly dependent on the electrode setup and the applied voltage and the signal frequency values. The length of the plasma jet increases when the applied voltage and/or its frequency increase, while the diameter at its end is significantly reduced when the applied signal frequency increases. For an applied voltage of 10 kV, the plasma jet diameter decreases from near 5 mm for a frequency of 10 kHz to less than 1 mm at a frequency of 50 kHz. This obtained size of the plasma jet diameter is very useful when the medical treatment must be processed in a reduced space.

**PACS 2008:**

52.50.Dg; 52.70.-m; 52.80.-s

## Background

Non-thermal atmospheric pressure plasma jets (NAPPJs) are widely studied because of their promising applications in several areas of industry as well as nano-medicine and biotechnology [1, 2]. The main advantages of this kind of atmospheric pressure non-equilibrium plasmas are their relatively easy implementation and their use at ambient conditions without any significant risks for the operator and/or the environment. Driven by different high-voltage signals (AC, DC, pulsed DC…) [3, 4] at low or high frequencies [5, 6], NAPPJs are able to produce electrons, ions, free radicals, and photons. Using adequate gas precursors, these reactive plasma species are useful in nano-medicine for bacterial inactivation [7], cancer treatment [8], blood coagulation, and injury healing process [9]. Most atmospheric plasma jets are based on dielectric barrier discharge (DBD) configurations, which have the benefit of avoiding glow to arc transition and homogenizing the electrical discharge. Although during these last few years several works have been devoted to the plasma jets technology and their applications, the dependence of the plasma jet characteristics on the electrical parameters of the excitation signal is still not well understood and/or controlled. In the present work, a homemade plasma jet reactor using argon gas as precursor has been developed. We present the results of some investigations about the effects of the electrical parameters of the excitation signal on the plasma jet geometry. This study is carried out in order to obtain a plasma jet with geometry suitable for localized treatments in reduced spaces.

## Methods

**Figure 1:**
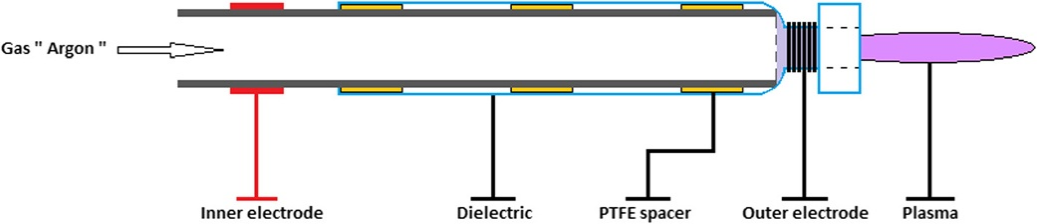
**Schematic representation of the plasma jet reactor.**

The schematic representation of our homemade non-thermal atmospheric pressure plasma jet reactor is represented in Figure 1. It is constituted by a quartz tube (quartz thickness = 1 mm) as a dielectric barrier, a stainless steel tube of 6 mm of external diameter and 1 mm of thickness inserted into the glass tube as an inner electrode, and a coiled tungsten wire of 0.5 mm of diameter placed around the neck of the glass tube as an outer electrode. In order to obtain a homogeneous DBD discharge, the stainless steel tube with a previously polished external surface was fixed at the center of the glass tube using PTFE spacers. The argon gas (99.99%) with a flow rate of about 8 l/min was injected through the inner electrode to the discharge area situated between the two electrodes. The volume of the discharge area was reduced by fixing the inner electrode very close to the outer one (the two electrodes were separated by a few millimeters), leading to less electrical power consumption (7 to 10 W) and to less energy dissipation, avoiding then the use of a cooling system. This reactor geometry prevents glow to arc transition due to charge propagation. The inner and the outer electrodes are connected to a Redline G2000 high-voltage pulse generator (Redline Technologies Elektronik GmbH, Baesweiler, Germany), able to deliver up to 20 kV peak to peak in a frequency range of 4 to 500 kHz. A Canon PowerShot SX220 HS camera (Canon Inc., Tokyo, Japan) was used to take photos of the created plasmas.

## Results and discussion

**Figure 2:**
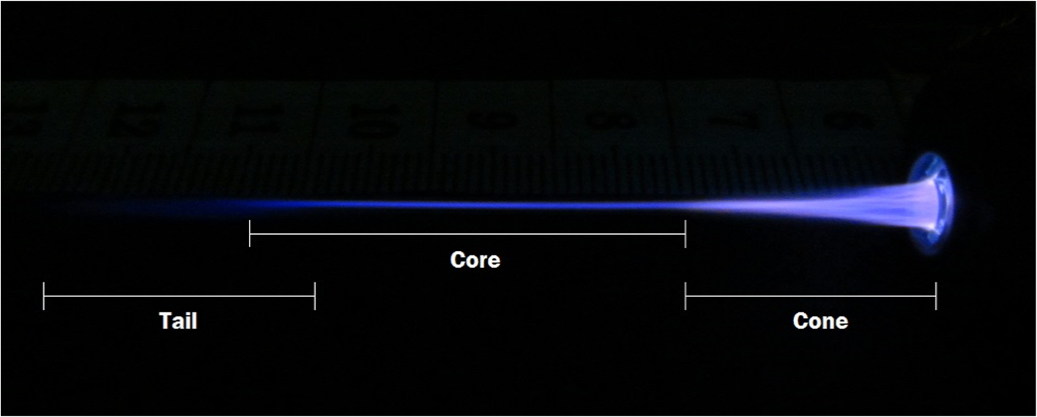
**Photo showing the three regions of a typical plasma jet created in argon gas (** ***V***_**pp**_ **= 10 kV;** ***f*** **= 50 kHz).**

A photo of a typical plasma jet created in argon gas is shown in Figure 2 for an applied voltage at the inner electrode of 10 kV and a frequency of 50 kHz. Three different regions are observed: the first one at the beginning of the jet with a conical shape, a second one at the middle of the plasma jet (the core), and a third one representing the tail of the jet. The diameter of the plasma jet is larger at the beginning than at its end. However, the geometry and the shape of the plasma jet were found dependent on the applied voltage and the signal frequency values. Depending on these electrical discharge parameters, the diameter of the plasma jet at the middle (core) and at its end can be varied from a few hundreds of micrometers to a few millimeters.

### Effect of applied voltage

**Figure 3:**
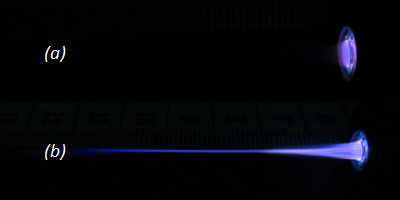
**Plasma jet length variation versus the electrode setup (** ***V***_**pp**_ **= 10 kV;** ***f*** **= 50 kHz). (a)** Inner electrode grounded and **(b)** outer electrode grounded.

A minimum value of about 6 kV is required to produce a stable plasma jet. Its length is dependent on the electrode setup. When the outer electrode was grounded, the plasma jet length reaches more than 60 mm, and when the grounded electrode was the inner one, the plasma length reaches only a few millimeters. Figure 3 shows clearly this behavior of the plasma jet for an applied voltage of 10 kV and a signal frequency of 50 kHz. These results are similar to those found by Shao et al. [10] on a plasma jet created in Ar gas by applying a voltage of 7.5 kV with a signal frequency of 17 kHz. The variation in the plasma jet behavior observed when the electrode arrangement is changed may be due to a difference between the amounts of the accumulated charges generated by the plasma discharge between the two electrodes. At the active electrode, the applied potential varies with time leading to an accumulation of charges during the first half period of the applied voltage and to an accumulation of the opposite type of charges during the next half period. These charges compensate the first ones, occurring then a partially or completely neutralizing charge process at this electrode. In contrary, as at the ground electrode the potential is fixed, an important amount of charge accumulated on the inner surface of the dielectric barrier (quartz tube), underneath this electrode, creating then a charge overflow. This charge overflow beyond the ground electrode leads to the development of a self-biasing voltage in this region. This promotes the charged species movement along the axis of the gas flow from the active electrode to the ground electrode and ignites plasma beyond the ground electrodes; an extensive glow discharge is created in this area and a more pronounced plasma jet length than that observed when the active electrode is the outer one is then obtained.

In the following, all presented results concern the outer electrode grounded configuration.

**Figure 4:**
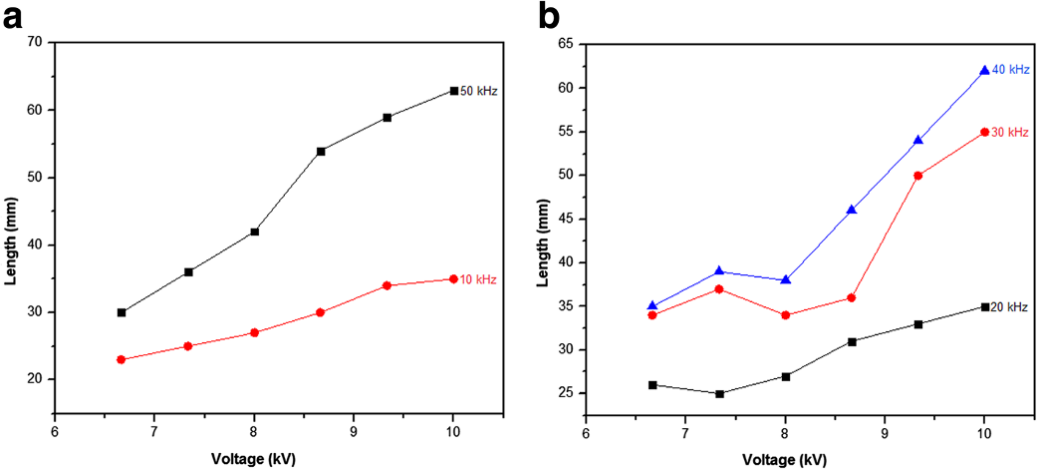
**Plasma jet length variation versus the applied peak to peak voltage at different signal frequencies (a, b).**

The effect of the applied voltage intensity on the plasma jet length is represented in Figure 4. For frequencies above 10 kHz and below 50 kHz (Figure 4b), two regions were observed: the first one between 6.6 and 8 kV where the plasma jet length increases slightly and the second one (beyond 8 kV) where the plasma jet length increases more promptly. The presence of a threshold-like voltage around 8 kV may be linked to a default in the matching network between the plasma generator and the impedance constituted by the created plasma jet and the plasma reactor [11, 12]. The electrical power transmitted to the plasma jet is more important when the matching network is well realized in the electrical system. In this case, after reaching the voltage value inducing the electrical breakdown of the argon gas, more energy is transferred into the discharge area. When the applied voltage increases, more energetic species are created and their pronounced energy allows them to penetrate deeper into the surrounding air, leading to the formation of an extended plasma jet. When the matching network is not well realized, the transferred energy is at its low level compared to the previous point. The created species are then less energetic and cannot penetrate into the surrounding air, leading to the creation of a shorter plasma jet.

### Effect of signal frequency

**Figure 5:**
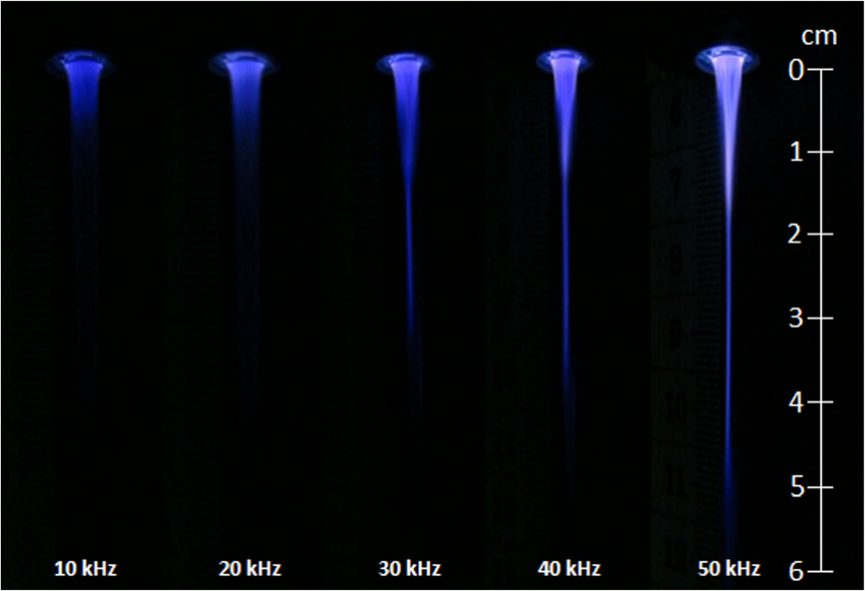
**Plasma jet length and shape variation for different signal frequencies (** ***V***_**pp**_ **= 10 kV).**

**Figure 6:**
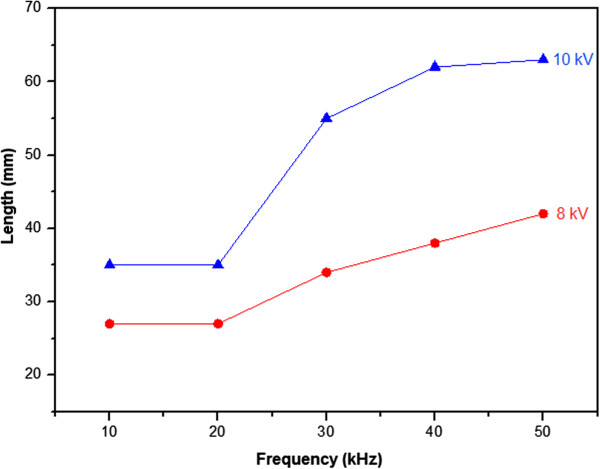
**Plasma jet length variation versus signal frequency.**

As it is shown in Figure 5, the signal frequency affects significantly the length and the shape of the plasma jet. For an applied voltage of 10 kV at a frequency of 10 kHz, a wide and hallowed plasma jet without the core zone and the tail region was created. As the frequency value increases from 10 to 20 kHz, the plasma jet becomes less hollowed and a bit brighter. At 30 kHz and beyond this frequency value, the plasma jet brightness is more important and the hallowed area decreases until it disappeared completely. The plasma jet becomes thinner and a pinch occurs leading to the formation of the core and the tail regions represented in Figure 2. This change in the plasma jet shape is accompanied by an increase of the plasma jet length as it is reported in Figure 6. Xiong et al. [13] have also reported this dependence of the plasma jet length on the signal frequency variation. Beyond 20 kHz, the plasma jet length increases significantly when the signal frequency is more pronounced. When the applied signal frequency increases from 20 to 50 kHz, the plasma jet length increases from 27 to 42 mm and from 35 to about 63 mm for an applied voltage of 8 and 10 kV, respectively. The diameter of the plasma jet in the core zone is reduced when the signal frequency increases, reaching a value at the middle of less than 1 mm for an applied voltage of 10 kV and a signal frequency of 50 kHz. The tail zone has a diameter slightly greater than that of the core zone. In this region considered as a turbulent region by Xiong et al. [14], the plasma species are less energetic and cannot penetrate deeper in the surrounding air. We have noticed that the length and diameter of this zone can be significantly reduced, until its disappearance, when the applied voltage is decreased from 10 to 6 kV. A thinner plasma jet of a shorter length constituted mainly by the core zone is then obtained. Such size of the plasma jet will be very beneficial for medical application when the treatment must be processed in a reduced space in the human tissue.

The effect of the signal frequency on the plasma jet length and shape can be explained by the variation of the spatiotemporal distribution of ions. As ions are heavier than electrons, when the signal frequency increases over 20 kHz, they cannot follow the signal variation and then become more static. They concentrate in the middle of the discharge area and the gas flow can project them farther, inducing an increase of the plasma jet length. This expansion of the plasma jet length is mainly due to an increase of the core length and in a smaller amount to that of the tail zone.

The shape of the plasma jet obtained at 10 kV by varying the signal frequency from 20 to 30 kHz is close to that observed by Nie et al. [15] and Li et al. [16] on the plasma jet of argon gas for an applied voltage and a signal frequency of 2.7 kV, 48 kHz and 2.6 kV, 40 kHz, respectively, and by Kim et al. [17] and Jiang et al. [18] on the plasma jet of helium gas for an applied voltage and a signal frequency of 1.2 kV, 50 kHz and 9 kV, 17 kHz, respectively.

## Conclusions

An argon-based non-thermal atmospheric pressure plasma jet has been created using DBD configuration. The dependence of the plasma jet geometry on the electrical parameters of the excitation signal and electrode setup has been investigated. It has been found that the shape and the length of the plasma jet are dependent on the electrode setup and the electrical parameters. The plasma jet spreads more towards the grounded electrode, and its length and shape can be controlled by varying the applied voltage and signal frequency values. A plasma jet with a core zone diameter of about a few hundreds of micrometers has been obtained. Such plasma jet size is very useful when the jet will be used in a reduced space and/or when the medical treatment must be well localized.

## References

[1] Walk RM, Snyder JA, Srinivasan P, Kirsch J, Diaz SO, Blanco FC, Shashurin A, Keidar M, Sandler AD (2013). Cold atmospheric plasma for the ablative treatment of neuroblastoma. J Pediatr Surg.

[2] Daeschlein G, Scholz S, Ahmed R, von Woedtke T, Haase H, Niggemeier M, Kindel E, Brandenburg R, Weltmann KD, Juenger M (2012). Skin decontamination by low-temperature atmospheric pressure plasma jet and dielectric barrier discharge plasma. J Hosp Infect.

[3] Xiong Q, Lu XP, Ostrikov K, Xian Y, Zou C, Xiong Z, Pan Y (2010). Pulsed dc- and sine-wave-excited cold atmospheric plasma plumes: a comparative analysis. Phys Plasmas.

[4] Li X, Di C, Jia P, Bao W (2013). Characteristics of a direct current-driven plasma jet operated in open air. Appl Phys Lett.

[5] Seo YS, Lee HW, Kwon HC, Choi J, Lee SM, Woo KC, Kim KT, Lee JK (2011). A study on characterization of atmospheric pressure plasma jets according to the driving frequency for biomedical applications. Thin Solid Films.

[6] Kim SJ, Chung TH, Bae SH, Leem SH (2009). Characterization of atmospheric pressure microplasma jet source and its application to bacterial inactivation. Plasma Process Polym.

[7] Guimin X, Guanjun Z, Xingmin S, Yue M, Ning W, Yuan L (2009). Bacteria inactivation using DBD plasma jet in atmospheric pressure argon. Plasma Sci Technol.

[8] Kim JY, Ballato J, Foy P, Hawkins T, Wei Y, Li J, Kim SO (2011). Apoptosis of lung carcinoma cells induced by a flexible optical fiber-based cold microplasma. Biosens Bioelectron.

[9] Raiser J, Zenker M (2006). Argon plasma coagulation for open surgical and endoscopic applications: state of the art. J Phys D Appl Phys.

[10] Shao XJ, Jiang N, Zhang GJ, Cao ZX (2012). Comparative study on the atmospheric pressure plasma jets of helium and argon. Appl Phys Lett.

[11] Hong Y, Lu N, Pan J, Li J, Wu Y, Shang KF (2013). Characteristic study of cold atmospheric argon plasma jets with rod-tube/tube high voltage electrode. J Electrostat.

[12] Shao XJ, Zhang GJ, Zhan JY, Mu HB (2011). Investigation on spurt length of atmospheric-pressure plasma jets. IEEE Trans Plasma Sci.

[13] Xiong Q, Lu X, Ostrikov K, Xiong Z, Xian Y, Zhou F, Zou C, Hu J, Gong W, Jiang Z (2009). Length control of He atmospheric plasma jet plumes: effects of discharge parameters and ambient air. Phys Plasmas.

[14] Xiong R, Xiong Q, Nikiforov AY, Vanraes P, Leys C (2012). Influence of helium mole fraction distribution on the properties of cold atmospheric pressure helium plasma jets. J Appl Phys.

[15] Nie QY, Ren CS, Wang DZ, Zhang JL (2008). A simple cold Ar plasma jet generated with a floating electrode at atmospheric pressure. Appl Phys Lett.

[16] Li X, Jia P, Yuan N, Fang T, Wang L (2011). One atmospheric pressure plasma jet with two modes at a frequency of several tens kHz. Phys Plasmas.

[17] Kim DB, Rhee JK, Gweon B, Moon SY, Choe W (2007). Comparative study of atmospheric pressure low and radio frequency microjet plasmas produced in a single electrode configuration. Appl Phys Lett.

[18] Jiang N, Ji A, Cao Z (2010). Atmospheric pressure plasma jets beyond ground electrode as charge overflow in a dielectric barrier discharge setup. J Appl Phys.

